# A reversible broad-spectrum antiviral targets the human V-ATPase V_O_ domain

**DOI:** 10.21203/rs.3.rs-9889115/v1

**Published:** 2026-06-03

**Authors:** Jimena Pérez-Vargas, Diana Bautista-Sánchez, Rebecca A. Oot, Connor A. H. Thompson, David E. Williams, Lianne Presley, Danielle G. Gordon, Annika Lea Schulz, Kassidy Knight, Siobhan Ennis, David Scheibner, Luka Krampert, Steven J. McArthur, Nidhi Kaushik, Syan Olver, Guang Gao, Jun Bae Park, Ivan Villanueva, Keryanne Gagnon, Alexander Nowakowski, Claire M. Brown, Leon H. Chew, Erin Knock, Brian Hetrick, Sandra Diederich, Soung-Hun Roh, Masahiro Niikura, Bryce Warner, Stephan Wilkens, Raymond J. Andersen, François Jean

**Affiliations:** University of British Columbia; University of British Columbia; State University of New York Upstate Medical University; University of British Columbia; University of British Columbia; University of British Columbia; University of British Columbia; University of British Columbia; State University of New York Upstate Medical University; Simon Fraser University; Federal Research Institute for Animal Health; University of British Columbia; University of British Columbia; Vaccine and Infectious Disease Organization, University of Saskatchewan; Vaccine and Infectious Disease Organization, University of Saskatchewan; University of British Columbia; Seoul National University; University of British Columbia; University of British Columbia; McGill University; McGill University; Stemcell Technologies; Stemcell Technologies; Virongy Biosciences, Inc; Federal Research Institute for Animal Health; Seoul National University; Simon Fraser University; Vaccine and Infectious Disease Organization, University of Saskatchewan; State University of New York Upstate Medical University; University of British Columbia; University of British Columbia

**Keywords:** broad-spectrum antiviral, host-directed antiviral, V-ATPase inhibition, pH-dependent viral entry, cladoniamide A

## Abstract

Increasing threats of viral disease underscore the urgent need for broad-spectrum antiviral drugs (BSADs). Host proteins utilized by human pathogenic viruses are key BSAD targets. The vacuolar-type H^+^-ATPase (V-ATPase) has been identified as a proviral factor for most pH-dependent enveloped viruses classified as pandemic threats. We report here the discovery of cladoniamide A (CA), a V-ATPase inhibitor with single-digit nanomolar antiviral activity and a high selectivity index (SI: 10^3^–10^4^) against human enveloped viruses [e.g., SARS-CoV-2 variants, influenza A viruses (H1N1, H5N1), respiratory syncytial virus, dengue serotypes 1–4, and Zika virus]. Transcriptome profiling, pH estimation assays, and V-ATPase bioassays indicate that CA interferes with V-ATPase-dependent acidification of the host endolysosomal network thus preventing viral entry. Using pseudoviruses derived from five pathogenic virus families, we confirmed that CA is an entry inhibitor BSAD. CryoEM revealed that CA inhibits the V-ATPase rotary motor by occupying unique binding sites in the membrane-embedded V_o_ motor.

Importantly, intranasal CA treatment in mice infected with influenza A H1N1 significantly reduced viral load in the lung by four log orders. Together, these findings pave the way for developing next-generation BSADs targeted at unique druggable pockets that enable the reversible pharmacological modulation of the human V-ATPase.

## Introduction

Emerging and re-emerging viral infections are a significant global health threat exacerbated by climate change, which increases the prevalence of zoonotic diseases and expands the range of disease-carrying vectors^1^. Throughout the 21^st^ century, numerous viral outbreaks have occurred, many linked to zoonotic or vector-borne viruses including influenza A H1N1 (2009), Ebola virus (EBOV, 2013–2016), Zika virus (ZIKV, 2015), as well as coronaviruses like SARS-CoV-1 (2003) and MERS-CoV (2012)^2^. The COVID-19 pandemic alone claimed approximately 14.9 million lives globally (WHO, 2022). Influenza A viruses (IAV) circulate annually and infect millions, especially children^3^, causing seasonal flu and periodic pandemics (e.g., H1N1 in 1918, 2009, and H3N2 in 1968)^4^. The H5N1 avian flu current outbreak raised concerns due to its spread to mammals, including cattle, thus increasing the risk of transmission to humans^5^. While vaccines have reduced the impact of these viruses, the continual emergence of new strains challenges vaccine efficacy. Antiviral drugs are therefore crucial for controlling outbreaks and reducing disease severity^6^. Most approved antivirals are direct-acting agents (DAAs), which target viral proteins directly^7^; however, their narrow spectrum of activity and propensity to induce drug resistance limit their effectiveness^8,9^. In contrast, host-directed antivirals (HDAs), which target stable host factors essential for viral replication, may offer a more sustainable solution^10,11^. As viruses continue to evolve and evade immunity from both natural infections and vaccines, there is an urgent need for broad-spectrum antiviral drugs (BSADs) that can target a wide range of viruses.

Proviral factors are host cellular proteins that are hijacked and repurposed by viruses to fulfill their life cycle and promote their propagation. One such protein complex is the vacuolar-type H^+^-ATPase (V-ATPase), a proton pump that primarily functions to acidify subcellular organelles. V-ATPase subunits were identified in iRNA, siRNA, and CRISPR screens as proviral factors across infection of different virus families^12–14^, underscoring their importance in promoting virus entry, trafficking, uncoating, and replication. Many enveloped viruses rely on endolysosomal pathways and the acidification of these compartments to trigger viral fusion or uncoating^15,16^. Additionally, V-ATPase activity influences antigen processing and presentation, autophagy, and vesicle-trafficking of immune molecules, thereby modulating host immune and antiviral responses^17^. Given its proviral role, V-ATPase represents a promising broad-spectrum antiviral target.

**Figure 1:**
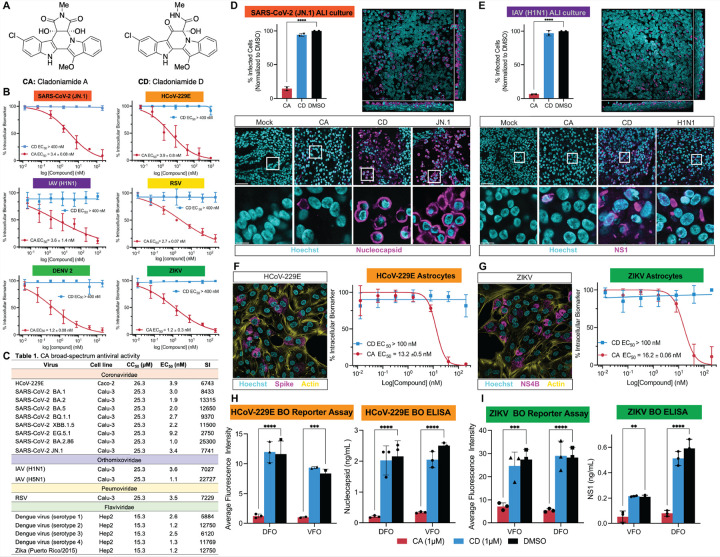
CA is a broad-spectrum nanomolar virus inhibitor. **A**) Chemical structures of active CA and inactive CD compounds. **B**) Dose-response curves of cells pretreated for 3 h with the indicated concentration of CA and CD before infection with SARS-CoV-2(JN.1), HCoV-229E, IAV(H1N1), RSV, DENV (serotype 2), and ZIKV for 48 h. Data acquisition was performed using the CellInsight CX7 HCS platform; EC_50_ values were determined using nonlinear regression analysis (n = 3). **C**) CA broad-spectrum antiviral activity table, SI (ratio CC_50_ /EC_50_) for all viruses tested. Fully differentiated air-liquid interface (ALI) cultures of primary human nasal epithelial cells treated with CA, CD, or DMSO before infection with SARS-CoV-2 JN.1 (**D**) and IAV/H1N1/2009 (**E**), staining for nucleocapsid or NS1(magenta), respectively, and nuclei (cyan). Quantification of infected cells is shown. Top and orthogonal views are shown. Images were acquired with a Leica STELLARIS 5 confocal microscope and 63X/1.40 oil objective lens. Representative images were selected from n = 2 independent experiments. Dose-response curves of astrocytes treated with the indicated concentrations of compounds and infected with HCoV-229E (**F**) and ZIKV (**G**) for 48 h. Immunofluorescence staining of human astrocytes for HCoV-229E spike protein, ZIKV NS4B protein, and actin filaments (F-actin). Images acquired using Leica STELLARIS 5 confocal microscope and 63X silicon oil immersion lens. Representative images selected from n = 3 independent experiments. DFO and VFO brain organoids were pretreated for 3 h with the indicated concentration of CA and CD before infection with HCoV-229E (**H**) and ZIKV (**I**). After 72 h of infection, the supernatants were collected, and reporter assays and ELISA were performed for each virus. Data are represented as mean (n=3). A two-way ANOVA was used to determine significant differences between infections. Only p-values less than 0.05 are shown (****p<0.0001, ***p ≤ 0.01).

Natural products (NPs) from plants, marine organisms, and microorganisms offer a promising source of new antiviral compounds. Derived from diverse sources, these compounds encompass the structural diversity that falls outside the scope of the chemical spaces found in synthetic chemical compounds, thus, harvestin the potential to act via mechanisms distinct from those of conventional therapies^18,19^. NPs have demonstrated antiviral activities and are expected to have good tolerability and minimal side effects^18,19^. Cladoniamides are examples of unprecedented tryptophan-derived alkaloid skeleton with an indolotryptoline core (Figure 1A). Cladoniamides A to G were isolated by our group in cultures of the bacterium *Streptomyces uncialis* harvested from the surface of the lichen *Cladonia uncialis* collected in British Columbia^20^. Reports on the bioactivity exhibited by this indolotryptoline alkaloid family include: submicromolar *in vitro* cytotoxicity against murine and human cancer cell lines^21,22^, induction of apoptosis in epidermal growth factor (EGF)-stimulated A431 cells that overexpress EGF receptor^23^, inhibition of the kinase CaMKIId^21^, inhibition of yeast and human V-ATPase^23,24^, and antimalarial activity^25^.

In this study, we discovered that cladoniamide A (CA), isolated from *Streptomyces uncialis*, exhibits nanomolar antiviral activity against respiratory-associated viruses [e.g., HCoV-229E, SARS-CoV-2 variants, IAV, and respiratory syncytial viruses (RSV)], mosquito-borne viruses (e.g., ZIKV and dengue viruses (DENV)), as well as emerging viruses like EBOV, Marburg virus (MARV), and Lassa virus (LASV). CA demonstrated nanomolar potency in both immortalized human cell lines as well as primary cells and brain organoids with high selectivity (SI) index values (10^3^ – 10^4^). Our pH bioassay demonstrates that CA is a reversible V-ATPase inhibitor that alkalinizes the host endo-lysosomal network, thereby blocking the entry of human enveloped RNA viruses. Cryogenic electron microscopy (CryoEM) analysis at ~3 Å confirms that CA binds to novel binding sites on the V-ATPase *c* subunits, acting as an HDA. In a mouse model, treatment with CA significantly reduced IAV H1N1 viral load in the lungs by four log orders. These virological, biochemical and structural insights are expected to accelerate the development of novel V-ATPase-targeted antivirals, offering a promising avenue for the treatment of numerous viral diseases.

### Viral infections blocked by CA

CA was selected for further validation as a new antiviral compound derived from the screening of a 373 pure NP library representing a large diversity of chemical scaffolds assembled from extracts of plants, fungi, bacteria, and marine sponges^20^. Compounds showing 80% or greater inhibition of SARS-CoV-2 infection with less than 20% cell loss were defined as potential candidates. Based on this criterion, CA was selected for further validation along with CD, an inactive analogue.

We next tested CA’s potency against a wide array of human enveloped RNA viruses including SARS-CoV-2 Omicron subvariants until the JN.1 strain, seasonal coronavirus HCoV-229E, low (H1N1) and high (H5N1) pathogenic IAV, RSV, the four serotypes of DENV, and ZIKV. Human immortalized cell lines were pre-treated with serially diluted NPs before virus infection, and relative quantification of infected cells was used to calculate half-maximal effective concentration (EC_50_) values, which ranged from 1 to 9.2 nM (Figures 1B–C **and S1**). The half-maximal cytotoxic concentration (CC_50_) values were also calculated and determined to be between 15.3 and 26.3 μM, orders of magnitude above the EC_50_ nanomolar range, for the different cell lines tested (Figures 1C **and S2A-D**). The SI was expressed as the ratio of CC_50_ over EC_50_ of CA. High values (10^3^–10^4^) were noted for all viruses tested (Figure 1C). A high SI is preferable if a drug is to be viewed as having a favorable safety profile in human cells^26^. These results suggest that CA has a potent nanomolar antiviral activity against multiple human enveloped RNA viruses with excellent efficacy-based therapeutic index values (SI>10^3^).

### CA antiviral activity in physiologically relevant cell-based systems

Immortalized cell lines are commonly used for virus isolation, propagation, and rapid antiviral screening. However, they have limitations in accurately replicating the physiological conditions encountered by the virus *in vivo*. In particular, the innate antiviral signaling pathways are often impaired in these models due to the process of cellular immortalization^27^. To better assess the antiviral activity of CA, we next evaluated its effects in more physiologically relevant models.

First, we focused on two respiratory viruses, SARS-CoV-2 and IAV using air-liquid interface (ALI) cultures of human airway epithelium. ALI models are considered the gold standard for respiratory disease modeling and drug screening because they closely mimic the human respiratory tract^28^. CA treatment resulted in an 85% and 95% reduction in SARS-CoV-2 and IAV infection, respectively, in the ALI system, versus infection in untreated (DMSO) or CD-treated controls (Figure 1D and 1E).

Next, we evaluated two neurotropic viruses, HCoV-229E^29^ and ZIKV^30^, using primary human astrocytes and brain organoids. Primary human astrocytes isolated from the cerebral cortex were pretreated with serially diluted CA or CD before infection. Via the quantification of the immunofluorescent staining of HCoV-229E spike S1 (Figure 1F) and ZIKV NS4B (Figure 1G), we calculated EC_50_ values. CA demonstrated potent antiviral activity, with EC_50_ values of 13.2 nM for HCoV-229E (Figure 1F) and 16.2 nM for ZIKV (Figure 1G).

To further explore CA’s effect on more complex models, we used pluripotent stem cell-derived brain organoids (BO), which replicate complex neural networks and functional properties of the human brain. These organoids are especially valuable for studying ZIKV pathogenesis in the context of congenital syndromes such as microcephaly. We used two types of brain organoids, ventral forebrain organoids (VFO) and dorsal forebrain organoids (DFO), differentiated for 170 days (HCoV-229E) and 63 days (ZIKV), respectively. BOs were pretreated with 1 μM CA or CD and then infected with HCoV-229E or ZIKV at MOI 1 for 3 h. After 72 h, the supernatants were collected to assess viral production. CA inhibited HCoV-229E and ZIKV infection in both types of brain organoids (VFOs and DFOs) as shown by reduced viral levels in the reporter assay using supernatants from CA-treated organoids versus untreated or CD-treated controls (Figure 1H, I). We confirmed these results using ELISA to detect HCoV-229E nucleoprotein (Figure 1H) and ZIKV NS1 (Figure 1I) in the supernatant of brain organoids. These findings are consistent with observations in human astrocytes and confirm the CA robust potency against HCoV-229E and ZIKV in primary human cells (Figure 1F–G). Our results strongly support the potential of CA as a BSAD that is effective not only in immortalized cell lines but also in more physiologically relevant systems such as ALI cultures, human astrocytes, and brain organoids.

### CA mode of action

**Figure 2:**
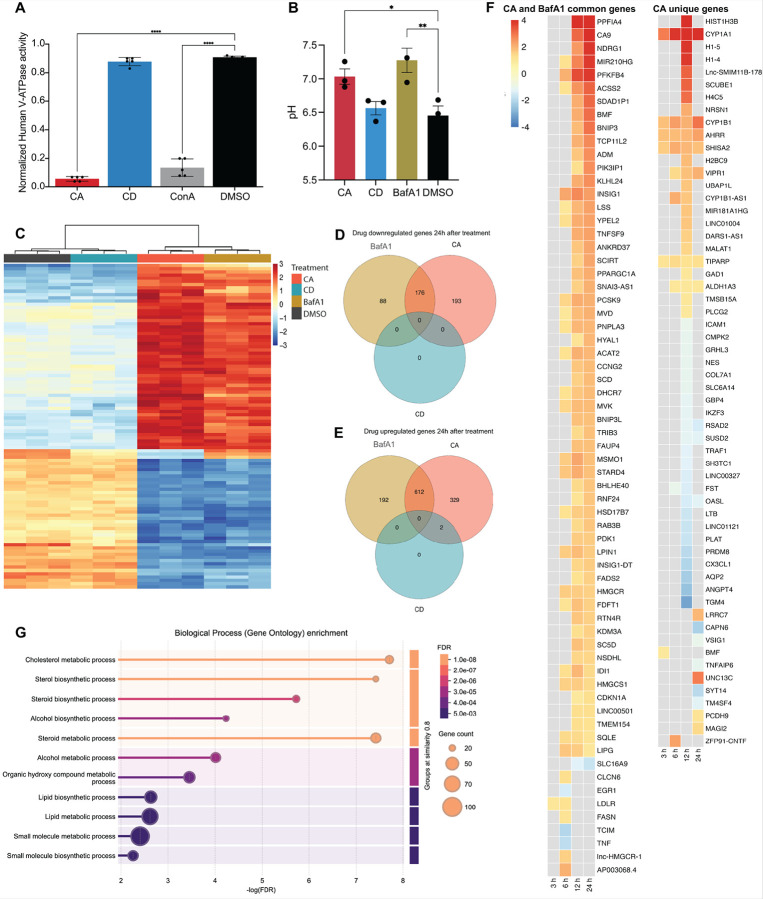
CA mode of action. **A**) Inhibition by CA, not CD, of the reconstituted human V-ATPase. 200 nM of CA (but not CD) inhibits the enzyme’s V-ATPase activity. **B**) CA prevents acidification of intracellular vesicles in human cells. The ratio of fluorescence intensity images from 488 nm versus 458 nm laser excitation was calibrated to extract pH values for individual vesicles. Data are represented as mean pH (n=3). A two-way ANOVA was used to determine significant differences between treatments. Only p-values less than 0.05 are shown (****p<0.0001, ***p ≤ 0.01, **p ≤0.01, *p ≤ 0.05). **C**) Heatmap displaying the top 100 significantly dysregulated genes across CA, CD, BafA1, and DMSO-treated samples at 24 h. Log2-fold change data were normalized to z-scores, and the top 100 genes were selected from the comparison between CA and DMSO treatments, based on the lowest p-value and p-adjusted values. **D**) Venn diagram showing the downregulated genes with a negative Log2-fold change in each of the treatments (CA, CD and BafA1) when compared to DMSO at 24 h. **E**) Venn diagram showing the upregulated genes with a positive Log2-fold change for each of the treatments (CA, CD and BafA1) when compared to DMSO at 24 h. **F**) Heatmap displaying the Log2-fold change of the commonly dysregulated genes in the CA and BafA1 treatment across time points (left). Heatmap displaying the Log2-fold change of the genes uniquely dysregulated in the CA treatment across time points (right). **G**) Biological process gene ontology (GO) enrichment for the CA and BafA1 commonly dysregulated genes at 24 h. Terms with a similarity higher than 0.9 were grouped. Rows were ordered by descending −Log10 false discovery rate (FDR).

After establishing the BSAD activity of CA, we sought to understand its molecular mechanism by identifying which cellular pathways and processes are affected, and thus potentially revealing common targets across different viruses. A previous study reported that a member of the cladoniamide targets the V-ATPase in yeast^24^. To validate our hypothesis that CA’s physiological effects are, at least in part, due to direct interaction with the proton-pumping V-ATPase, we evaluated the effect of CA and CD on the function of the yeast (*Saccharomyces cerevisiae*) and human enzymes *in vitro*(Figures 2A **and S2E**). Purified yeast and human V-ATPase complexes reconstituted in lipid nanodiscs^31,32^ were treated with CA or CD, and V-ATPase activity was measured using an ATP-regenerating enzyme-coupled assay^33^.We used the well-characterized V-ATPase inhibitors, bafilomycin A1 (BafA1), and concanamycin A (ConA), as controls. BafA1 and ConA are known to inhibit proton pumping by targeting the *c* subunit (ATP6V0c) of the V-ATPase V_o_ subcomplex, thereby blocking lysosomal acidification^31–33^. CA significantly reduced the V-ATPase activity in both yeast and human V-ATPase to levels comparable to ConA, whereas inactive CD had no significant effects (Figures 2A **and S2E**). These results support the hypothesis that CA’s antiviral activity is at least partially mediated through inhibition of V-ATPase activity.

We next measured the pH of intracellular vesicles in human lung cell lines Calu-3 and A549 (Figures 2B **and S3**). Cells were treated with CA, CD, or BafA1 and imaged using an LSM 710 confocal microscope. Fluorometric pH assays were conducted using a ratiometric pH sensor and a confocal imaging system^34^. We found that both CA and BafA1 inhibited vesicle acidification with an average vesicle pH above 7.0 (Figures 2B **and S3A-C**). To further investigate pH dynamics, we monitored intracellular pH changes over time using the pH-sensitive dye acridine orange (AO), a weak base (pK_a_ = 9.65) that accumulates in acidic compartments^35,36^. AO fluorescence intensity decreased following treatment with CA or BafA1, indicating elevated vesicle pH and lack of AO accumulation. Notably, the effect of CA was reversed after 6 h, and the granular pattern of AO-loaded acidic compartments was recovered. In contrast, BafA1 inhibition of acidification persisted for more than six hours (**Figure S3D-G**).

To better understand the CA mechanism at the molecular level, we performed time-resolved transcriptomic profiling in Calu-3 cells treated with CA, CD, or BafA1 and compared the results to those of control cells (Figure 2C). We found that CA downregulated 369 transcripts, 176 of which overlapped with those downregulated by BafA1 (Figure 2D). Furthermore, CA upregulated 943 transcripts, of which 329 are unique to CA, 612 are shared with BafA1, and only two are shared with CD (Figure 2E–F). Notably, both CA and BafA1 showed enrichment of cholesterol, steroid metabolism, and biosynthesis pathways (Figure 2G); CA and CD shared upregulation of pathways involved in tryptophan metabolism.

Together, these results highlight CA’s ability to reversibly block vesicle acidification and modulate distinct gene expression programs. In addition, the transcriptomic profile suggests that the antiviral activity of V-ATPase inhibition by CA may extend beyond a direct effect of altered pH to include other processes influenced by enzyme function, like the cholesterol metabolic process^37^.

### CryoEM structure of CA-bound V_o_ subcomplex

**Figure 3:**
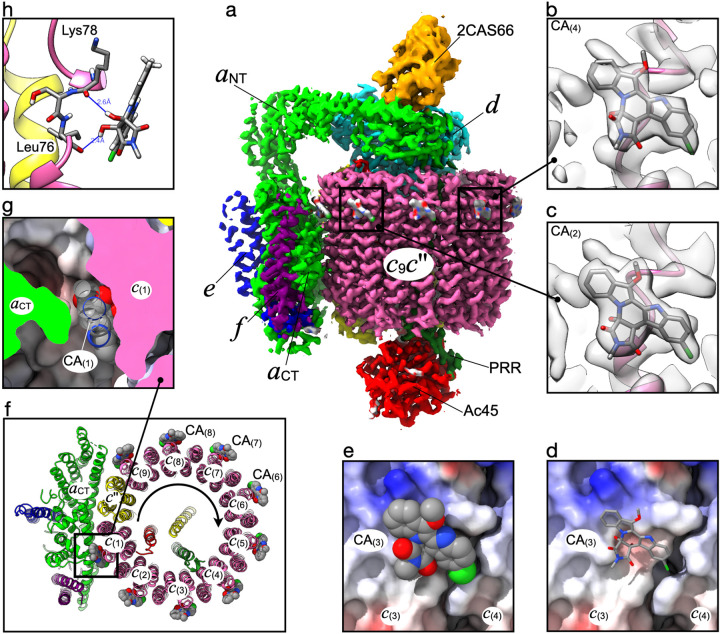
CryoEM of CA bound human V_o_. **A**) CryoEM map of *a*4-V_o_ in lipid nanodisc. V_o_ was immunocaptured using a camelid nanobody (Nb; 2CAS66) raised against the N-terminal domain of V_o_ subunit *a* isoform *a*4 and supplemented with 100 μM cladoniamide A before vitrification. **B, C**) EM density for CA molecules bound to *c* subunits *c*_(2)_ and *c*_(4)_. **D, E**) Stick and space-fill representation of CA molecule bound to *c*_(3)_ rendered in electrostatic surface. **F**) View towards the membrane showing nine CA molecules bound to the V_o_ *c* subunits. **G**) CA molecule bound between *c*_(1)_ and *a*_CT_. **H**) CA molecules form H-bonds with backbone carbonyl oxygens of leucine 76 and lysine 78 of the *c* subunits.

V-ATPase is a rotary motor enzyme composed of two subcomplexes: a cytosolic ATPase called V_1_ and a proton channel referred to as V_o_. ATP hydrolysis on V_1_ drives rotation of an integral ring of proteolipid *c* subunits, which carries protons between two aqueous half-channels located in the V_o_ subunit *a*. A prior study in *Schizosaccharomyces pombe* indicated that CA may bind the proteolipid *c* subunits of the V_o_^24^. To determine the binding site for CA on the human enzyme, we determined the cryoEM structure of lipid nanodisc-reconstituted human V_o_ vitrified in the presence of a ~two-fold molar excess of CA (100 μM; two-fold molar excess considering stoichiometry of nine *c* subunits per complex) based on the estimated concentration of the preparation used for cryoEM (~5 μM). From a dataset of 3,438 images, we were able to obtain a map of the CA-bound complex at an overall resolution of ~3 Å (Figures 3A, **S4**, and **Table S1**). In addition to the previously observed densities for V_o_ subunits *a*,*c*,*c*”, *d*,*e*,*f*, Ac45, PRR, and tightly bound (glyco)lipids, the map also showed prominent densities located at the periphery of the *c* subunits near the cytosolic side of the *c*-ring (Figure 3A–C). The densities match the shape of CA and are well resolved for all nine of the *c* subunits (**Figure S5**). The interaction between CA and the *c* subunits appears to be mostly driven by shape complementation with the single chloride moiety bridging the gap to an adjacent *c* protomer (Figure 3D–E). Of note, CA binding occurs near residues V60, V118, M131 (residue numbering of the human enzyme) that, when mutated, confer resistance to CA in *S. pombe*^24^. As has been observed previously for autoinhibited V_o_ from yeast and rodents, human isoform *a*4 containing V_o_ is halted in rotary state 3. In this configuration, the proteolipid isoform *c*” is in the *a*_CT_:*c*-ring interface with the conserved glutamate forming a salt bridge with the conserved arginine in *a*_CT_ (Figure 3F). ATP hydrolysis-driven rotation of the *c*-ring past *a*_CT_ in active holo V-ATPase is required for proton translocation across the membrane. In the CA-bound complex, however, the CA molecule bound to *c*_(1)_ (or any of the nine CA molecules) is predicted to clash with residues of *a*_CT_ (Figure 3G), thus providing a mechanistic rationale for why CA binding inhibits holoenzyme activity. In addition to hydrophobic shape complementation, CA binding to subunit *c* appears to be stabilized by two hydrogen bonds between CA’s two hydroxyl groups and backbone carbonyls of L76 and K78 (Figure 3H).

### CA activity impact on viral entry

**Figure 4:**
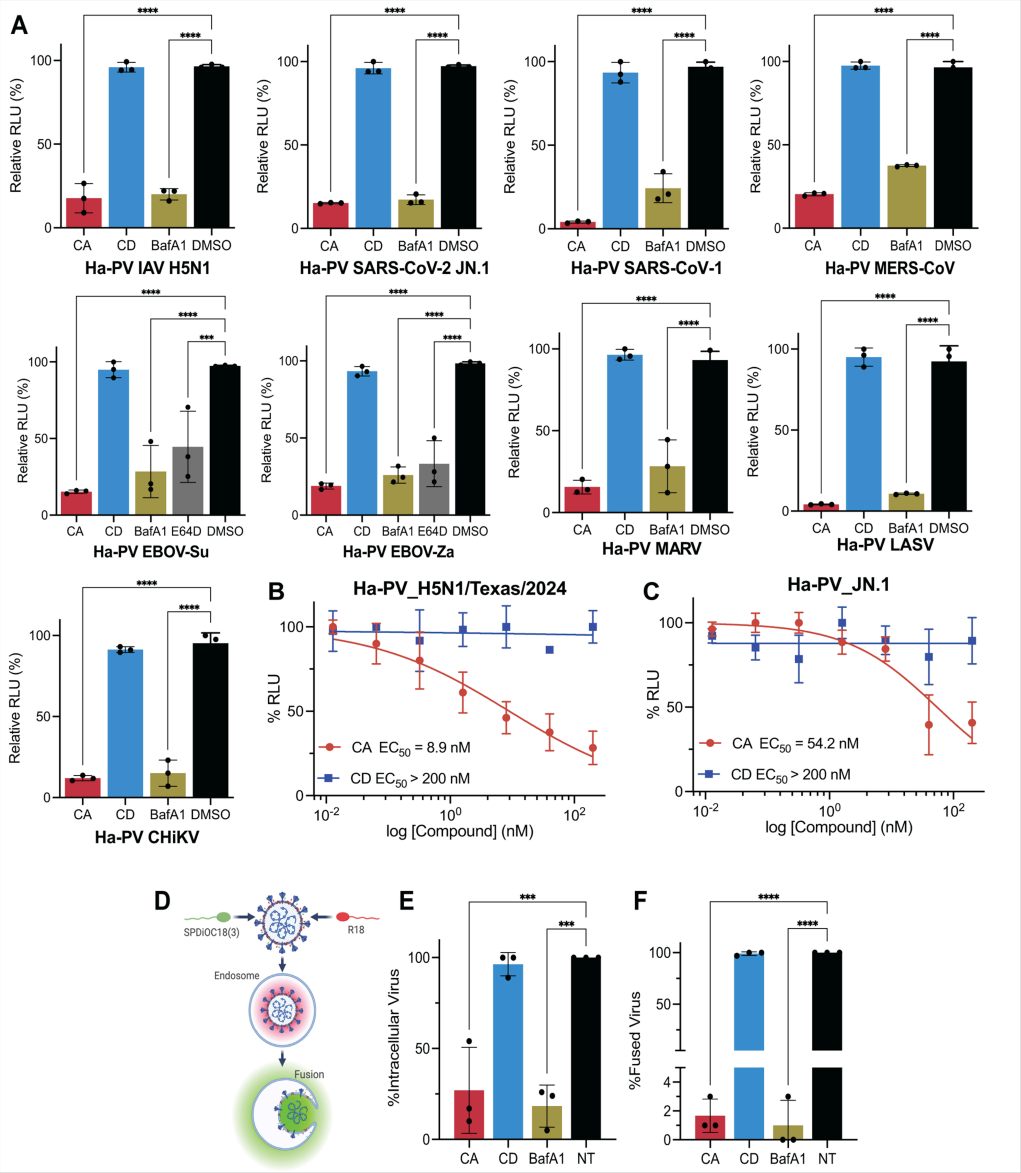
Entry inhibition of pseudoviruses and viral fusion assays. **A**) Cells were pretreated for 1 h with 200 nM of inhibitors before treatment for 18 h with Ha-PV-(Luc). Pseudovirus entry inhibition was quantified by luciferase assay. Data were normalized to quantify luciferase with DMSO-treated pseudovirus. Dose-response curves of cells pretreated for 1 h with the indicated concentration of CA and CD before 18 h treatment with (**B**) Ha-PV(Luc)-H5N1/Texas/2024 or (**C**) HA-PV(Luc)- SARS-CoV-2/JN.1. Pseudovirus entry inhibition was quantified by luciferase assay. EC_50_ values were determined using nonlinear regression analysis. The GraphPad Prism 10^™^ (GraphPad Software, Inc.) nonlinear regression fit modelling variable slope was used to generate a dose-response curve [Y = Bottom + (Top-Bottom)/(1+10^((LogIC_50_-X)*Hillslope)] constrained to top = 100, bottom = 0. **D)** Fusion assay scheme, **E**) the intracellular HCoV-229E virus, and **F**) HCoV-229E fused virus were quantified after CA treatment. Data are represented as mean (n=3). A two-way ANOVA was used to determine significant differences between infections. Only p-values less than 0.05 are shown (**** p < 0.0001; ***p ≤ 0.01).

V-ATPase activity can affect various stages of the viral life cycle. To determine whether CA-mediated inhibition of V-ATPase impacts the entry of the virus into the cell, we used the hybrid alpha-pseudovirus (Ha-PV) containing all the proteins forming the viral particle but lacking the ability to replicate (**Figure S6A-B**). We used Ha-PVs for SARS-CoV-2 JN.1, IAV H5N1, SARS-CoV-1, MERS-CoV, EBOV Sudan (Su), EBOV Zaire (Za), MARV, LASV, chikungunya virus (CHIKV), and Nipah virus (NiV). We found that CA can block 75–85% of the Ha-PV entry except for NiV (Figures 4A **and S6C**). However, when we performed an EC_50_ curve with the Ha-PV for SARS-CoV-2-JN.1 and H5N1/Texas/2024 (Figure 4B–C), we obtained values 15 or 8 times greater, respectively, than with the virus (Figures 1B **and S1**). This suggests that life cycle stages other than entry are affected for some viruses. In conclusion, this data centred the entry stage as a key viral life cycle stage at which CA inhibits infection.

For viruses that hijack the endolysosomal system, entry encompasses endocytosis and fusion. To further dissect the role of CA in these entry steps, we performed a fusion assay in human primary astrocytes infected with HCoV-229E. This virus uses the endosomal pathway for cell entry, and its fusion process relies on acidic pH environments. HCoV-229E virions were dually labeled with two lipophilic fluorescent dyes, R18 (red) and SP-DiOC_18_(3) (green). The virions exhibit only red fluorescence when entering the cell; however, after fusion has occurred, green fluorescence coming from the fused virions can be detected (see Methods section). Thus, red fluorescence signals incoming virions (intracellular virus), while green fluorescence marks successful viral fusion events (Figure 4D). When primary astrocytes were treated with the acidification inhibitors, CA and BafA1, both the red and green intensity readouts were lower when compared to the untreated group. In CA-treated cells, signal was reduced by ~75% and >95% respectively, as opposed to CD-treated cells, which showed no difference to untreated group (Figure 4E–F). Both acidification inhibitors inhibited entry and fusion of HCoV-229E; the latter may be a consequence of its pH neutralization activity (Figure 4E). Overall, these data suggest that CA can affect virus entry into host cells via endocytosis.

**Figure 5:**
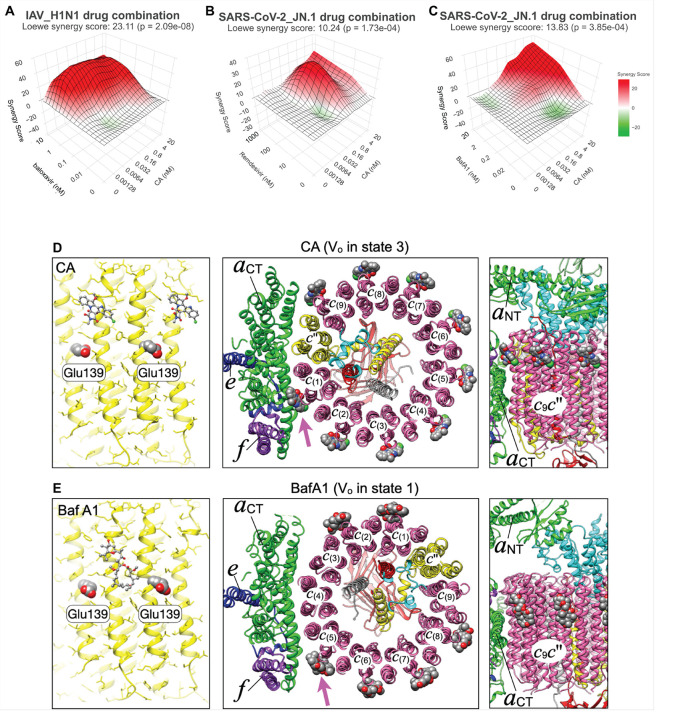
Synergistic inhibition of CA and locations of CA and BafA1 V-ATPase binding pockets. Combined treatment of CA and (**A**) baloxavir in IAV H1N1, (**B**) remdesivir or (**C**) BafA1 in SARS-CoV-2-infected Calu-3 cells was used for synergy analysis using the open-source web application SynergyFinder. 3D visualization of synergy maps for CA with baloxavir (**A**), remdesivir (**B**), or BafA1 (**C**) was generated using the Loewe additive model. The surface is color-coded: red indicates synergistic interactions, and green indicates antagonistic interactions (n = 2). **D-E**) Comparison of CA and BafA1 binding sites. **D**) CA bound to human *c* subunits (PDBID: 9DET) and **E**) BafA1 bound to human and bovine (central & right panel; PDBID: 7KHR) *c* subunits, respectively.

V-ATPase activity is essential for many cellular processes, and thus, toxicity could be a concern. Multi-targeted treatments lead to therapeutic benefits by reducing drug toxicity, enhancing efficacy and avoiding monotherapy resistance. To explore the therapeutic benefits of CA when used in combination with viral replication inhibitors, we next determined whether CA could act synergistically when combined with IAV or SARS-CoV-2 DAAs (baloxavir and remdesivir, respectively). We infected Calu-3 cells pretreated with either CA, baloxavir, or remdesivir as a single treatment or in combination^19^. First, the dose-response curves of inhibition for single and combined treatments indicate that the inhibitory effect of the CA-based drug combinations is more robust than that a single compound alone (**Figure S7**). We then used four different synergy reference models^38^ to analyze the potential synergistic action between CA and baloxavir or remdesivir (Figures 5A–B, **S7**). The drug interaction synergy scores and combination efficiencies were calculated with the open-source web application SynergyFinder, where scores above 10 are interpreted as synergistic^38^. Combining CA with baloxavir or remdesivir shows broad synergistic activity against IAV H1N1 and SARS-CoV-2 JN.1 as indicated by calculated synergy scores exceeding 10 (Figures 5A–B, **S7**). Also when BafA1 was used in combination with CA for SARS-CoV-2 JN.1 infection, we found a synergistic effect (score above 10) blocking the viral infection (Figures 5C, **S7**). CA binding does not significantly impact the proteolipid’s structure as revealed when comparing the CA-bound structure to the structure obtained in the absence of inhibitors (**Figure S8**). Moreover, CA binding is significantly closer to the headgroups of the lipid bilayer (Figure 5D–E) compared to the V-ATPase-specific inhibitor BafA1, which binds the *c* subunits near the conserved glutamate residues (E139 in human V-ATPase) mid bilayer^39^. The closer proximity of CA’s binding site to the aqueous cytosol may (in part) explain the synergistic activity observed when combining CA and BafA1 (Figure 5D–E).

### Bioavailability, general toxicity, and antiviral efficacy of CA in small animal models

**Figure 6:**
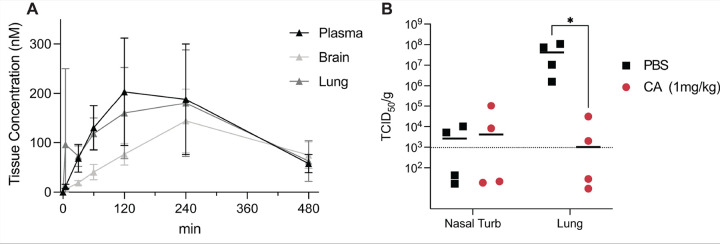
CA PK studies and efficacy in a mouse model of IAV H1N1. **A**) Mice were administered CA, 1 mg/kg, intranasally. The C_max_ and half-life values were determined in plasma, brain and lungs. **B**). BALB/c mice were treated twice, 2 h pre-infection with influenza A/PR/8/34 (H1N1). TCID_50_ (50% tissue culture concentration dose) on day 3 post-infection. n = 4 mice per group. Statistical significance was assessed by Kruskal-Wallis test (*p ≤ 0.05).

Pharmacokinetic (PK) measurements were performed to test the stability of CA *in vivo* andto evaluate the therapeutic potential.Initial results from murine PK (1 mg/kg) studies determined maximum concentration (C_max_) of 400 nM and half-life values of 202 minutes in the blood/plasma and brain of mice treated with intranasal (IN) CA (Figure 6A). The C_max_ is 200 times the half-maximal effective concentrations required for the CA to inhibit viral infection (Figure 1C). To evaluate the general toxicity of CA, the compound was dosed IN at 24 h intervals with a dose of 1 mg/kg in male Balb/c mice. Mortality and clinical signs were collected and analyzed at 0.5 h, 2 h, 4 h, and 6 h after the first administration of the compound and thereafter daily for two consecutive days. The mice demonstrated no lethality and no external signs of toxicity within two dosing days and one post-dosing day. These results validate CA’s drug potential.

To determine whether compound CA can inhibit IAV replication and reduce disease *in vivo*, we next conducted a mouse challenge experiment where mice were pre-treated 2 h with either CA or PBS as a control (Figure 6B). Mice were administered an IN dose of PBS or CA and then challenged two hours later with the PR8 strain of H1N1 influenza, a mouse-adapted IAV that results in lethal disease^40^. Four mice per group were euthanized on day 3 post-challenge to examine viral burden in the nasal turbinate and lungs. Viral titers were low or undetectable in the nasal turbinates across both groups, likely due to the challenge method of virus instillation deep into the lungs. However, lung titers were four-log higher in PBS-treated mice than in the CA-treated group (Figure 6B). Overall, our data suggest that intranasal treatment of mice with CA can significantly reduce IAV H1N1 replication in the airway.

## Discussion

In this study, we report on CA, a potent NP with nanomolar antiviral activity against respiratory-associated viruses (e.g., IAV, RSV, SARS-CoV-2, SARS-CoV-1, and MERS-CoV), mosquito-borne viruses (e.g., ZIKV, DENV and CHIKV), and emerging viruses EBOV, MARV, and LASV. The nanomolar potency of CA against various viral infections in immortalized cell lines, as well as primary astrocytes, ALI culture and brain organoids without detectable toxicity, yields a SI of greater than 10^3^. In mouse models, CA significantly reduced influenza A H1N1 viral load in the lungs by four log orders. We demonstrated that CA’s antiviral activity depends on a pharmacophore that includes a closed ring structure and a vicinal diol structure, which appears to be a major binding site that is not present in the inactive analogue CD^20^. We showed that CA, but not CD, effectively inhibits functionally reconstituted yeast and human V-ATPase activity, and we determined the structure of CA-bound human V-ATPase proton channel subcomplex. In regards to its action mechanism, the monitoring of pH changes in Calu-3 cells in the presence of CA using a pH-sensitive ratiometric indicator and AO, and demonstrated that CA is a reversible pH inhibitor, contrary to the prolonged inhibitory effect of BafA1. However, the identification of the transcriptional upregulation of cholesterol and steroid metabolism and biosynthesis pathways shared by BafA1 and CA, suggest that CA’s role may go beyond a direct effect on the endolysosomal pH. The unique tryptophan pathways activated under CA and CD treatment. The upregulation of CYP1A1 and CYP1B1 is a consequence of their nature as tryptophan metabolites, which are aryl hydrocarbon receptor (AhR) ligands, a transcription factor that regulates gene expression and controls imbalances in tryptophan, also acting as an immunity modulator^41^. However, no antiviral effect was ever detected under CD treatment. These data suggest that CA represents a potential broad-spectrum natural product antiviral candidate against emerging viruses and human viruses of pandemic concern.

CA could potentially exhibit broad-spectrum activity across tissue and cell types because its target, V-ATPase, is ubiquitously expressed. V-ATPase is a highly conserved multi-subunit ATP-driven proton pump responsible for acidifying intracellular compartments including endosomes, lysosomes, synaptic vesicles, and the *trans*-Golgi network. V-ATPase’s proton-pumping function is critical for a multitude of essential biological processes including pH homeostasis, protein trafficking and degradation, endocytosis, and neurotransmitter loading^42^. V-ATPase is a rotary nanomotor composed of two subcomplexes: a peripheral V_1_-ATPase and a membrane integral V_o_ proton channel^43^. ATP hydrolysis on the V_1_ drives rotation of the *c*-ring, which results in proton translocation across the *c*-ring:*a*_CT_ interface in the V_o_^44,45^. In our studies, the CA-bound V_o_ subcomplex of the human V-ATPase showed CA binding to the *c* subunits in a manner that likely interferes with the rotation of the *c*-ring along *a*_CT_, thereby blocking proton transport. Notably, the CA-bound V_o_ reveals that CA and BafA1 occupy different binding sites, which may explain the synergistic effect of the two inhibitors when used in combination and the difference in their reversibility profiles. BafA1 binds primarily via hydrophobic interactions, anchoring itself deeply within the membrane^39^, whereas CA depends more on hydrogen bonding and shape complementation.

CA inhibits the infection of several human enveloped RNA viruses that enter the host cell via endocytosis and release their viral contents into the cytoplasm in a pH-dependent process. Cargo transport through the endolysosomal system occurs through vesicle maturation. Cargo transport through the endolysosomal system relies on vesicle maturation, a process accompanied by a pH drop (early endosomes: 6.5 → lysosomes: 4.0) that relies on the increased activity of V-ATPases^46^. The entry and fusion of many viruses in the endolysosomal system allows their pericentric movement, capsid/core priming, and the proton- and/or protease-dependent activation of their fusion machinery. The latter process relies on enzymes from acidic compartments, such as cathepsins and furin, which function optimally at low pH environment and thus, require V-ATPase activity. Additionally, many viral fusogens undergo conformational changes that depend on proton binding/low pH^47^. In this study, we confirmed that CA binds the V-ATPase’s *c* ring, which compromises the proton-pump mechanism and results in endolysosomal pathway deacidification. We further validate the endolysosomal-dependent viral entry, particularly fusion, as a compromised stage by CA. Nevertheless, V-ATPase plays a crucial role in other cellular processes such as receptor recycling and the assembly and egress of some viruses. For example, previous studies reported that increased endosomal pH derived from V-ATPase inhibition resulted in altered endolysosomal trafficking and receptor recycling disturbances^37^.

As revealed by our transcriptional profiling, inhibiting the endolysosomal acidification by blocking the V-ATPase with CA and BafA1 results in increased transcription of genes involved in the biosynthesis and metabolism of cholesterol and steroids. pH regulation and the aforementioned pathways are intrinsically linked. Extracellular cholesterol uptake and distribution rely on the endolysosomal system, where it is transported as a component of membrane-bound vesicles or bound to sterol transfer proteins that move cholesterol between organelles (i.e., late endosomes and lysosomes to the ER)^48^. Proper cholesterol and lipid distribution is essential for maintaining the membrane structure, resulting in its permeability and fluidity, efficient transport within the secretory and exosomal pathways, vesicle and viral budding from the plasma membrane^49,50^. Thus, modulating cholesterol and lipid metabolism could be the key to new antiviral therapies.

V-ATPase is highly conserved and ubiquitously expressed, thus positioning it as a broad-effective and drug-resistant host target for viral infections. A pan-antiviral strategy that targets cholesterol homeostasis could harness a deliberate accumulation of cholesterol within the endosomal system. Other small molecules that inhibit the activity of V-ATPase have been identified: The first and most prominent being BafA1. Concerns about BafA1 toxicity have stalled its application as an antiviral despite its potential. A key difference with CA is the complex formation with the V-ATPase *c*-ring and the localization of CA binding pocket, which enables CA to exhibit a faster reversible effect than BafA1 in terms of blocking the proton translocation and allowing the cells to reestablish their function once they clear the viral infection. CA significantly reduce viral load in the mouse model. Also, the use of CA in combination with other treatments can be administered at lower doses, which could reduce treatment side effects. Using CA as an antiviral to block V-ATPase-mediated endosomal acidification may represent a promising approach for antiviral therapy. This is due to several factors: (I) the revelation of a new target pocket, (II) its pivotal role in facilitating viral entry, (III) its highly conserved target nature, (IV) targeting cholesterol homeostasis is a promising antiviral therapy, (V) its limited susceptibility to drugresistance mutations, (VI) its broad-spectrum activity against various viruses, and (VII) the potential for combination therapy to minimize adverse effects.

## Methods

### Cell lines, antibodies and inhibitors

All cells were from the American Type Culture Collection (ATCC) and were cultivated according. HEK293 (CRL-1573^™^) were maintained in Dulbecco’s Modified Eagle’s Medium (DMEM). Calu-3 (HTB-55^™^), HT1080 (CCL-121^™^), Hep-2 (CCL-23^™^), and Caco-2 (HTB-37^™^) cells were maintained in Eagle’s Minimum Essential Medium (EMEM), with 10% and 20% fetal bovine serum (FBS), respectively. Vero E6 cells (CRL-I1586^™^), for generating viral stocks, were maintained in MEM, with 10% FBS, 1 mM C_3_H_3_NaO_3_, and 0.1 nM non-essential amino acids. VeroE6/TMPRSS2 cells (JCRB 1819) were maintained in MEM with 5% FBS, penicillin, and streptomycin (100 IU and 100 μg/ml, respectively). Air-liquid interface (ALI) cultures of primary human nasal epithelial cells [MucilAir^™^ Pool, MP0012, Epithelix Sàrl (Geneva, Switzerland)] were reconstituted using a mixture of cells isolated from 14 donors, and maintained in MucilAir^™^ medium (Epithelix). Primary astrocytes isolated from human cerebral cortex at 21 weeks of gestation were purchased from ScienCell Research Laboratories and maintained in astrocyte medium (Cat#1801) with 1% astrocyte growth supplement (Cat#1852), 2% FBS, and penicillin (100 U/mL)/streptomycin (100 μg/mL) solution (Cat#0503). STEMCELL Technologies kindly provided human-induced pluripotent stem cell-derived ventral and dorsal forebrain organoids. The primary antibodies, kindly provided by GeneTex, were the following: SARS-CoV-2 nucleocapsid (GTX635679), ZIKV NS1 (GTX634158) and NS4B (*G*TX133321), DENV NS4B (GTX124250), IAV hemagglutinin (GTX127357) and NS1 (GTX 125990), and RSV F protein (GTX40697). H5N1 NP antibody HB65 was kindly provided by the laboratory of Bernd Köllner, FLI. dsRNA antibody (Scicons, J2–1904); HCoV-229E Spike S1 Polyclonal Antibody (PA5120720), Hoechst 33342, Alexa Fluor 647 phalloidin (A22287), goat anti-mouse HRP (A16066), goat anti-mouse IgG Alexa Fluor 488 (A11001), and goat anti-rabbit IgG Alexa Fluor 555 (A21428) were obtained from ThermoFisher. Dr. Raymond J. Andersen produced CA and CD^20^. BafA1 (11038) and baloxavir (3512–50) from Cayman Chemical Company, remdesivir (1809249-37-3), and concanamycin A (C9705) from Millipore-Sigma. DMSO was used as a vehicle for all drugs.

### Viruses and cell infection

All SARS-CoV-2 infections were carried out in Biosafety Level 3 (BSL3) facilities (either UBC FINDER or SFU BIO3) following Public Health Agency of Canada and UBC FINDER or SFU BIO3 regulations (UBC BSL3 Permit #B20–0105 and SFU Permit #361–2021). Omicron variants BA.1 (BC-SFU-OM6), BA.2 (provided by Dr. Mel Krajden, BC Centre for Disease Control, BC, Canada), BA.5 (NR-58616), BQ.1.1 (NR-58976), XBB.1.5 (NR-59104), EG.5.1 (NR-59503), BA.2.86 (NR-59638), and JN.1 (NR-59693) were obtained by BEI Resources. All infections with HCoV-229E (ATCC^®^ VR-740), RSV (ATCC^®^ VR-26PQ), IAV/H1N1 (ATCC^®^ VR-1894), ZIKV (ATCC^®^ VR-1843), and the four serotypes of DENV (kindly provided by Dr. Drebot) were carried out in a Biosafety Level 2 (BSL2) facility following Public Health Agency of Canada regulations (UBC BSL2 Permit # B17–0024). A/chicken/Ger-NW/AI04286/2022 H5N1 was obtained from the national reference lab for avian influenza viruses of Timm Harder at the Friedrich-Loeffler Institut (Germany), and infections were carried out in BSL3. Cells were seeded in 96-well plates the day before infection (10^4^ cells/well); virus stocks were diluted in cell-specific media to a multiplicity of infection (MOI), according to each virus, without antibiotics^18,50,51^. For H5N1 staining NP antibody (HB65) and secondary HRP goat anti-mouse antibodies were used. Finally, TrueBlue peroxidase substrate (SeraCare, 5510–0030) was added to make infected cells visible and analyzed via a microscope. Ventral forebrain organoids (VFO) and dorsal forebrain organoids (DFO) were differentiated for 60 and 170 days for ZIKV^51^ and HCoV-229E infection, respectively. In a 96-well round-bottom plate, VFO and DFO were pre-treatment for 3 h with 1 μM of compound (CA or CD), and infected at MOI of 1 for 3h. Supernatants were collected to perform reporter assay and ELISA at 72 h and 120 h post-infection for ZIKV (Biofront, Zik-NS1-Ek-192) and HCoV-229E (Genetex, GTX535824), respectively. Intracellular dose-response (EC_50_ values) against each virus was determined by pre-treating cells for 3 h with serially diluted compounds followed by viral infection for 48 h^11,19,52^.

### Infection of ALI-cultured primary human nasal epithelial cells (HNEpC)

ALI cultures (basal chamber) were pretreated with compounds for 3 h. IAV H1N1 and SARS-CoV-2 JN.1 virus stocks were diluted in MucilAir^™^ media to a MOI of 1 and 0.2, respectively. A 1:1 mixture of diluted virus and the compound was added to the apical chamber for 2 h, after apical chamber was washed with phosphate-buffered saline (PBS). Cells were fixed with 4% formalin for 1 h at RT 48 (IAV/H1N1) and 72 (SARS-CoV-2/JN.1) hours post-infection (hpi). The membranes were peeled from the inserts and washed with PBS for 10 min, blocked with 2% normal goat serum, 1% BSA, 0.1% cold fish skin gelatin, 0.1% Triton X-100, 0.05% TWEEN^®^ 20, 0.05% NaN_3_ in PBS for 1 h at RT. Primary antibodies were in buffer (1% BSA, 0.1% cold fish skin gelatin, 0.05% NaN_3_ in PBS). Secondary antibodies were incubated in the dark. Membranes were mounted onto Fisherbrand^™^ Superfrost^™^ microscope slides (Fisher Scientific; 1255015) using Fluoromount-G^™^ Mounting Medium (ThermoFisher).

### Fusion assay

Viral labeling was performed by adding a mixture of dyes [1 μM of SP-DiOC18 (3) and 1.9 μM of R18] to a 100 μL HCoV-229E viral stock (2.9×10^7^ PFU/mL). The labeled virus was incubated with agitation 1 h at RT in the dark. These virions only exhibit red fluorescence when entering the cell due to self-quenching and fluorescent resonance energy transfer (FRET) occurring from SP-DiOC18 (3) to R18. Once the virions fuse, the distance between the fluorophores increases, resulting in the dequenching of SP-DiOC18 (3) and the loss of FRET effect. Thus, virions appear green after successful fusion events. Human primary astrocytes were seeded at 10^4^ cells/well in a 96-well plate 24 h in advance^53–55^. Cells were pretreated with compounds for 3 h, and then the labeled virus was added, followed by 6h incubation. The plates were stained with Hoechst (1:3333) for 20 min, and the media were replaced with Live Cell Imaging Solution [LCIS, Invitrogen, A59688DJ] before imaging in the CX7 CellInsight HCS. Quantification was performed by analyzing the Mean Object Spot Total Intensity and normalizing the results of the experimental groups against the results from the mock-infected groups.

### Ha-PV(luc) pseudovirus entry inhibition assay

Cells were seeded (2×10^4^ cells/well) in 96-well white plates. A 2 h pre-treatment was performed with compounds at 200 nM. A normalized preparation of Ha-PV (luc) (from Virongy Bioscience) was then added to wells. Treatment proceeded for 18 h, and luminescence was detected on the SpectraMax Gemini XS spectrofluorometer (Molecular Devices, LLC) between 550–570 nm with a read time of 0.3 seconds/well^56^. Data were normalized to the amount of luciferase with only the pseudovirus.

### Nanoparticle tracking analysis (NTA)

The size distribution and particle number per millilitre of Ha-PV were measured using NanoSightPro (Malvern Panalytical). Ha-PV samples were diluted 1:25 in PBS. NTA was performed by light scattering with a capture duration of 500 frames. Data were collected and analyzed using NS Xplorer software. Each Ha-PV sample was measured five times (**Figure S6B**).

### Drug combination

Two drug combinations were tested using five-fold and ten-fold serial dilutions of CA and BafA1, baloxavir or remdesivir EC_50_, respectively^19,52,57^. Different dilutions of the two compounds were mixed and added to Calu-3 cells for 3 h before being infected with IAV H1N1 or SARS-CoV-2 JN.1 for 48 h. The expected responses were calculated based on four different synergy reference models using the open-source web application SynergyFinder^38,58,59^.

### Intracellular pH

Cells were seeded (2×10^4^ cells/well) in 96-well white plates. A 3 h pretreatment was performed with 200 nM compounds, and 0.1% DMSO as a control. The compounds were removed, and the media were changed for further incubation for 3, 6, 12, or 24 h. The 0 h time point plate was washed with HBSS before incubating with acridine orange (1:4000) and Hoechst 33342 at 1.5 μg/mL in LCIS for 20 min at 37°C. The plate was washed with HBSS, and LCIS was added to all the wells. Quantification was performed using CX7 CellInsight HCS, assessing the intensity of acridine orange spots per object detected. Finally, all samples were normalized to the DMSO control group.

### Intracellular vesicular pH

Cells were seeded on 8-well chambers (ibidi, cat: 80807) 24 h before the experiment. Cells were incubated with 1 mg/mL FITC-labelled 70 kDa dextran (ThermoFisher, cat: D1821) in serum-free media for 2 h at 37°C (pulse phase). Following the pulse phase, a pH-dosed buffer (pH 4.5, 5.0, 5.5, 6.0, 6.5, 7.0, 7.5, 8.0) supplemented with 5 μg/mL nigericin (Sigma-Aldrich, cat: N7143–5MG) was added to each well to construct a pH calibration curve. pH calibration solutions were prepared using either MES (pH ≤ 5.5) or HEPES (pH ≥ 6) buffers. HEPES and MES buffers comprised 10 mM NaCl, 135 mM KCl, 10 mM glucose, 100 μM CaCl_2_, 1 mM MgCl_2_, and 20 mM of either MES or HEPES. After the pulse phase, the samples were washed with PBS and treated with 200 nM of compound (CA, CD or BafA1) and DMSO 0.1% for 3 h. Later, cells were imaged on the LSM 710 confocal microscope mounted to an Axio Observer motorized inverted microscope (Carl Zeiss). Live cells were imaged using a stage-mounted incubation chamber (Live Cell Instrument). Images of 1024 × 1024 pixels, 12-bit depth, 4-line average, and 1.58 μs pixel dwell time were captured using a 20x PLAN APOCHROMAT, NA 0.8 objective lens Two image tracks with the same emission wavelengths were created for 2% power and 650 V PMT gain for 488 nm and 3% power and 750 V PMT gain for 458 nm excitation from a 25 mW argon-ion laser. The detector pinhole for both tracks was set to 2 Airy units, and digital offset to 10. The transmitted PMT was set to a gain of 250 V and was enabled to provide a brightfield image of cells. Three images of cells were captured per well.

Images were processed using FIJI software. The global intensity background was corrected by subtracting the modal pixel value from each pixel in the image. The background-corrected images were processed in Imaris (Bitplane, version 9.72), where vesicles were segmented as “spots.” The XY diameter was set to 1.24 μm, and local background subtraction was enabled. A threshold was set to detect the vesicles in each image. “Spots of different sizes” were enabled with region growing type being set as local contrast, and region growing diameter set as diameter from volume to ensure vesicles of different sizes were detected. The threshold for varied vesicle size was then set manually through visual inspection for each image. Vesicle mean intensity and max intensity were exported. Mean intensity ratios of 488 nm/458 nm fluorescence signal were calculated for each vesicle. To exclude near-zero and saturated intensity values, vesicles with a maximum 488 nm value above 4000 and a 458 nm mean value below 5 were excluded. Mean intensity ratios were plotted in the GraphPad Prism 11^™^ (GraphPad Software, Inc.), and pH values from samples were interpolated using the pH calibration curve modeled to a Boltzmann non-linear equation. Vesicle ratio data was normalized for each experiment to the maximal vesicle ratio calculated for DMSO treated control cells.

### Purification of human holo V-ATPase and Vo subcomplex

Human V_o_ complex in lipid nanodiscs (HsV_o_ND) was purified as previously described^60^. The lipid nanodisc-reconstituted V_o_ complex was concentrated by pull-down using a biotinylated anti-*a*4 Nanobody (Nb). NB generation and characterization will be described in detail elsewhere. Briefly, glycerol density gradient fractions containing V_o_ complex were incubated with a six-fold molar excess of Nb at RT for 2.5 h before the addition of 20 μl of streptavidin beads, and further incubation for 1.5 h. Beads were pelleted at 1,500 × *g* for 1.5 min at 4 °C, washed in 4 ml TBSE (20 mM Tris, 150 mM NaCl, 0.5 mM EDTA, pH 7.2, 1 mM DTT), and eluted by HRV-3C protease cleavage of the biotinylation tag overnight in a volume to obtain a final HsV_o_ND concentration of ~2 mg/ml.

### V-ATPase activity assay

V-ATPase activity was measured in the presence and absence of inhibitors (200 nM) using a coupled enzyme assay as previously described^60^. Briefly, 1 ml of the assay containing 50 mM HEPES, pH 7.5, 25 mM KCl, 0.5 mM NADH, 2 mM phosphoenolpyruvate, 5 mM ATP, and 30 units each of lactate dehydrogenase and pyruvate kinase was preheated to 37°C and supplemented with 4 mM MgCl_2_. The assay was started by adding ~5–10 μg V-ATPase, and the change in absorbance at 340 nm was monitored using a temperature-controlled cuvette holder in a Varian CARY 100 Bio UV-Visible spectrometer in kinetics mode. As controls, enzyme activity was measured with and without the addition of the specific V-ATPase inhibitor concanamycin A (ConA, 200 nM) and DMSO (vehicle) alone.

### CryoEM

For cryoEM structure determination, Nb-bound HsV_o_ND was incubated with 5% v/v 2 mM CA in DMSO (final CA concentration 100 μM) for 0.5 h at 4°C before vitrification on freshly glow discharged AuFlat 1.2/1.3 grids in liquid ethane using a self-built plunger. Grids were examined in a ThermoFisher Glacios transmission electron microscope with a Falcon IVi direct detector. A dataset of 3,438 movies was analyzed using the CryoSPARC^™^ package of programs^61^; CryoSPARC algorithms were used for rapid unsupervised cryo-EM structure determination^61^ (**Figure S6**; **Table S1**). The final 3-D reconstruction (consensus map) was calculated from 144,439 particle images to an overall resolution of 3 Å (resolution range 2.7–3.2 Å). The dataset was further sorted into five classes, and class 1, which showed the best density for the cytosolic side of the V_o_ (Nb bound *a*_NT_; 31,410 particle images), was then merged with the consensus map to generate a composite map using Phenix^62^.

### Model building

Model building used the V_o_ portion from coordinates for human *a*4 containing V-ATPase (7UNF; https://doi.org/10.1038/s41467-022-30899-z) and the *a*1-containing enzyme (6WM2; https://doi.org/10.1016/j.molcel.2020.09.029). Coordinates and restraints for CA were generated from the SMILES string CN1C(=O)C2(C3=C(C4=C(C5=CC=CC=C5N4C2(C1=O)O)OC)NC6=C3C=C(C=C6)Cl)O using the Grade2 server^63^. Atomic models were placed into the cryoEM density map using rigid body fitting in Chimera^64^ followed by manual building and automated real space refinement using Coot^65^ and Phenix, respectively.

### RNA extraction.

Calu-3 cells were seeded on six-well-plates 24 h before the compound treatment. A 3 h pretreatment was performed with 250 nM CA, CD, 100 nM BafA1, and 0.1% DMSO. Compounds were removed, and samples were incubated with fresh media until sample collection at the designated time points (3, 6, 12, and 24 h post-treatment). After the three biological replicates were collected, the total number of samples was randomly divided into groups for RNA extraction to avoid the batch effect. RNA extraction was performed with RNeasy Protect Kit (cat #74624). Samples were stored at −80°C until quantification and quality assessment using the NanoDrop One and Agilent 2100 Bioanalyzer, respectively. Subsequently, 80% of the samples tested were found to have a concentration higher than 100 ng/μL, and all of them had an RNA Integrity Number (RIN) between 8.8 and 10.

### Next generation sequencing and analysis

NGS was performed at the UBC Bioinformatics and Sequencing Consortium (BSC) using the NextSeq 2000 P3 platform. Each sample was subjected to 20 million reads (read length of 61 bp and paired-end reads). Adaptor trimming was performed using Cutadapt v2.3 before alignment with STAR 020201 to the reference human genome (GRCh38). Gene counts and differential expression analysis were conducted using FeatureCounts (v2.0.1) and DESeq2 (v.1.44.0), respectively. Analysis in the R programming language was done with the support of the UBC Life Sciences Institute Bioinformatics Facility. We used the Bioconductor Pathview packages for data visualization. The heatmaps were generated using pheatmap (v.1.0.12), and the Venn diagrams were created using ggvenn (v.0.1.10). The pheatmap showing the dysregulation across drug treatments at 24 h was done using z-score values of the top 100 genes with the lowest p and p-adjusted values when comparing CA and DMSO-treated samples collected 24 hours post-treatment. The heatmap showing the CA and BafA1 commonly dysregulated genes across time points showed all commonly dysregulated genes for time points 3, 6, and 12 h. For the 24 h timepoint, only commonly dysregulated genes that were dysregulated at any of the previous timepoints are shown. The heatmap showing the genes exclusively dysregulated for CA was done by comparing the CA and BafA1 datasets. The biological process gene ontology enrichment used the STRING database (v.12) with the subset of CA and BafA1 commonly dysregulated genes (788) and the universe of detectable genes in our experiment (15,342). The maximum FDR shown was set to 0.05, and the minimum gene count in the network was set to 2.

### CA bioavailability and general toxicity animal studies

The PK and general toxicity studies were conducted by Bienta/Enamine (Kyiv, Ukraine) All *in vivo* protocols were approved by Bienta’s Animal Care and Use Committee in adherence with the European Convention for the Protection of Vertebrate Animals used for Experimental and other Scientific Purposes (1986, ETS 123, ISSN 0070–105X). The CA concentrations in samples were determined using highperformance liquid chromatography-tandem mass spectrometry (HPLC-MS/MS). Mass spectrometric analysis was performed using an API 3000 (triple-quadrupole) instrument from AB Sciex (Canada) with an electro-spray (ESI) interface. The data acquisition and system control were performed using Analyst 1.6.3 software from AB Sciex.

### Animal model study

Viral challenge study was performed at the Vaccine and Infectious Disease Organization (VIDO) at the University of Saskatchewan. Experiments were approved by the institutional Animal Review and Ethics Board under Animal Use Protocol number 20250010 under guidelines provided by the Canadian Council on Animal Care. All infectious work performed with the influenza A virus was conducted under CL-2 conditions. To test the efficacy of CA *in vivo*, 4- to 6-week-old BALB/c mice were purchased from Charles River Laboratories and allowed to acclimate for at least seven days. Mice were treated with either PBS or CA diluted in PBS for a final dose of 1 mg/kg in 50 μL final volume (n = 4 mice per group). Treatments were performed by instillation of PBS or the drug compounds in PBS via the intranasal route. Mice were anesthetized with inhaled isoflurane, and the treatments were instilled through the nares (25 μL per nare). The mouths of the mice were held closed to allow for inhalation of the compounds into the lungs. Two hours following the first treatment, mice were inoculated with 100 TCID_50_ of the PR8 strain of H1N1 influenza A (A/Puerto Rico/8/1934 H1N1) via the same intranasal instillation method. Mice were administered the viral dose in 50 μL of plain media. Mice were weighed and monitored daily for clinical signs of disease as per the approved human intervention point protocol included in the Animal Use Protocol. Mice reaching humane endpoints were euthanized via inhaled isoflurane and cervical dislocation. On day 3 post-inoculation, the mice (n = 4 mice per group) were euthanized, and then blood, nasal turbinate, and lung samples were collected for viral titration.

### Examination of viral burden

TCID_50_ (50% tissue culture concentration dose) assays were performed to measure viral titers in tissues. Samples were frozen at −80°C for storage. Each sample was weighed and placed in plain MEM and then homogenized with a 5-mm stainless steel bead in a tissue lyser (Qiagen). Homogenates were clarified by centrifugation at 1500g 10 min, and ten-fold serial dilutions of tissue homogenates were made in MEM supplemented with 0.1% BSA and a 2X P/S. Dilutions were added in triplicate to 100% confluent MDCK cells in 96-well plates, washed with PBS, and then placed in MEM, 0.1% BSA and 1 μg/mL TPCK trypsin. Cytopathic effects were read on days 3 and 4 post-infection. TCID_50_ values per gram of tissue were calculated using the Reed-Muench method^66^.

## Supplementary Material

Supplementary Files

This is a list of supplementary files associated with this preprint. Click to download.

- CAsupplementary20251128lowR.pdf

## Data Availability

All data supporting the findings of this study are available within the paper and it Supplementary Information. The cryoEM density maps and atomic coordinates have been deposited in the Electron Microscopy Data Bank and the Protein Data Bank, respectively. The accession numbers are as follows: PDBID 9DET and EMD-46798 for atomic coordinates and composite map; EMD-46795 and EMD-46796 for consensus and class 1 maps, respectively. Transcriptome data were deposited into the GEO DataSets database under accession number GSE313871 and are available at the following URL: https://www.ncbi.nlm.nih.gov/geo/query/acc.cgi?acc=GSE313871 Ratiometric pH images are available upon request.
